# Ultrafast optical control of surface and bulk magnetism in magnetic topological insulator/antiferromagnet heterostructure

**DOI:** 10.1038/s41598-022-16205-3

**Published:** 2022-07-15

**Authors:** Peiwen Liu, Chris Eckberg, Lei Pan, Peng Zhang, Kang L. Wang, Gunter Lüpke

**Affiliations:** 1 Department of Applied Science, The College of William and Mary, Williamsburg, VA 23187 USA; 2 Department of Electrical Engineering, University of California, Los Angeles, CA 90095 USA; 3 Fibertek Inc, Herndon, VA 20171 USA; 4 DEVCOM Army Research Laboratory, Adelphi, MD 20783 USA; 5 DEVCOM Army Research Laboratory, Playa Vista, CA 90094 USA

**Keywords:** Condensed-matter physics, Ferromagnetism, Magnetic properties and materials, Spintronics, Topological matter, Materials for devices, Electronic devices, Information storage

## Abstract

Optical control of the magnetic properties in topological insulator systems is an important step in applying these materials in ultrafast optoelectronic and spintronic schemes. In this work, we report the experimental observation of photo-induced magnetization dynamics in the magnetically doped topological insulator (MTI)/antiferromagnet (AFM) heterostructure composed of Cr-(Bi,Sb)_2_Te_3_/CrSb. Through proximity coupling to the AFM layer, the MTI displays a dramatically enhanced magnetism, with robust perpendicular magnetic anisotropy. When subjected to intense laser irradiation, both surface and bulk magnetism of the MTI are weakened by laser-induced heating of the lattice, however, at the surface, the deleterious heat effect is compensated by the strengthening of Dirac-hole-mediated exchange coupling as demonstrated by an unconventional pump-fluence-dependent exchange-bias effect. Through theoretical analyses, the sizes of exchange coupling energies are estimated in the MTI/AFM bilayer structure. The fundamentally different mechanisms supporting the surface and bulk magnetic order in MTIs allow a novel and distinctive photo-induced transient magnetic state with antiparallel spin configuration, which broadens the understanding of the magnetization dynamics of MTIs under ultrashort and intense optical excitation.

## Introduction

Magnetic topological insulators (MTIs) have generated considerable interest as promising potential building blocks for novel spintronic and quantum computing devices. Due to the topological protection afforded by the time-reversal symmetric Z_2_ invariant, topological insulators (TIs) feature electronic and magnetic properties robust against local perturbations. Introduction of magnetic order into a TI breaks the time-reversal symmetry (TRS) of the topological Dirac states. Such TRS breaking opens a gap across the node of the surface Dirac band and lifts the spin and valley degeneracy, coalescing the typically metallic massless Dirac fermions into a massive state and precipitating novel phenomena such as the quantum anomalous Hall effect^1^ (QAHE) and magnetoelectric effect^2^. In practice, there are two effective ways to generate magnetic ordering in TIs. One is incorporating magnetic impurities into the TI crystal structure through chemical doping^1,3^, and another approach is via proximity effect, achieved by interfacing the TI with a ferromagnetic or antiferromagnetic layer in a planar heterojunction^4,5^. In the former case, long-range magnetic order is believed to be supported by two primary mechanisms. The first is van Vleck valence-electron-mediated ferromagnetism, wherein localized *p* electrons couple to the Cr 3d-orbital moment^1^. The other is the Ruderman–Kittel–Kasuya–Yoshida (RKKY) interaction where the ordering between magnetic dopants is maintained by coupling to itinerant fermions^6–8^. Due to the electronic localization of inverted bands caused by the large spin–orbit coupling in TI samples, van Vleck mechanism is believed to be the dominant source of spontaneous magnetic order in the bulk of magnetically doped MTIs^1^. The surface magnetization in MTIs, meanwhile, is believed to feature strong contributions from RKKY coupling between the magnetic dopants and delocalized Dirac fermions in the topological surface state^6–9^.

Although research on bulk and surface magnetic properties in MTIs has expanded understanding of these systems tremendously^10–14^, details of how the RKKY and van Vleck mechanisms coexist and cooperate to sustain magnetism in doped MTIs remain unsettled. Particularly, while the dynamic magnetic properties of these materials can generate insight into the origin of magnetism in these systems, few ultrafast optical studies have been reported on MTIs to date. Even though ultrafast optical manipulation of magnetism has been well-studied in conventional magnetic materials where magnetism is mediated via the exchange interaction^15–18^, the ultrafast optical control of magnetism in TI materials is novel and requires its own exploration. We will note, however, that there are several reports investigating the response of either surface or bulk electrons to photon excitation in nonmagnetic TI materials^19–22^, notably, including the observation of very short-lived (200 fs) magnetization in TI materials under ultrafast photoexcitation^23–25^.

In this study, we use transient magneto-optical Kerr effect (MOKE) spectroscopies to investigate the magnetization dynamics in Cr-(Bi,Sb)_2_Te_3_ (CBST)/CrSb heterostructure. By performing pump-modulated MOKE measurements with a variety of pump fluences we observe a transition from a uniformly magnetized state to a photo-induced dynamical exchange-spring magnetic structure indicative of a decoupling between the MTI surface and bulk magnetizations. As a consequence of this decoupling, an unconventional, pump-dependent exchange bias effect is observed at high pump intensities. We further record the evolution of pump-modulated transient MOKE hysteresis loops at different pump-probe delays, elucidating different characteristic lifetimes for the photo-induced bulk and surface magnetic orders, confirming the excited carrier (in)sensitivity of the latter (former). We demonstrate the coexistence and interplay of both the bulk van Vleck coupling and surface RKKY coupling in the MTI system. Our results push forward the understanding of surface and bulk magnetism under ultrashort optical excitation.

## Results

Experiments have been carried out on Cr-doped (Bi,Sb)_2_Te_3_ (10 nm)/CrSb (10 nm) heterostructure epitaxially grown on epi-ready semi-insulating GaAs(111)B substrates. The in-plane lattice constant a = 4.122 Å of AFM CrSb is close to that of Cr-doped (Bi,Sb)_2_Te_3_ ($a=$ 4.262–4.383 Å)^5,26^, which enhances the feasibility for growing such MTI/AFM heterostructure by molecular beam epitaxy. To avoid the three-dimensional growth mode of CrSb with low crystal quality, a 2-nm buffer layer of undoped (Bi, Sb)_2_Te_3_ is grown on the GaAs substrate before the growth of CrSb. After the film growth, a 2 nm Al layer is evaporated on the sample surface in-situ at room temperature to prevent contamination and oxidation. In the MTI layers, the Bi:Sb ratio is set to be 0.3:0.7, and the Cr-doping concentration is 13%, which places the Fermi level within the surface band gap, resulting in an insulating character. The high crystal quality and clean interface of the Cr-doped (Bi,Sb)_2_Te_3_/CrSb heterostructure, characterized by high-resolution transmission electron microscopy on a different sample but grown under similar parameters in the same chamber^5^, make it possible to probe the surface and interface through MOKE spectroscopy.

**Figure 1:**
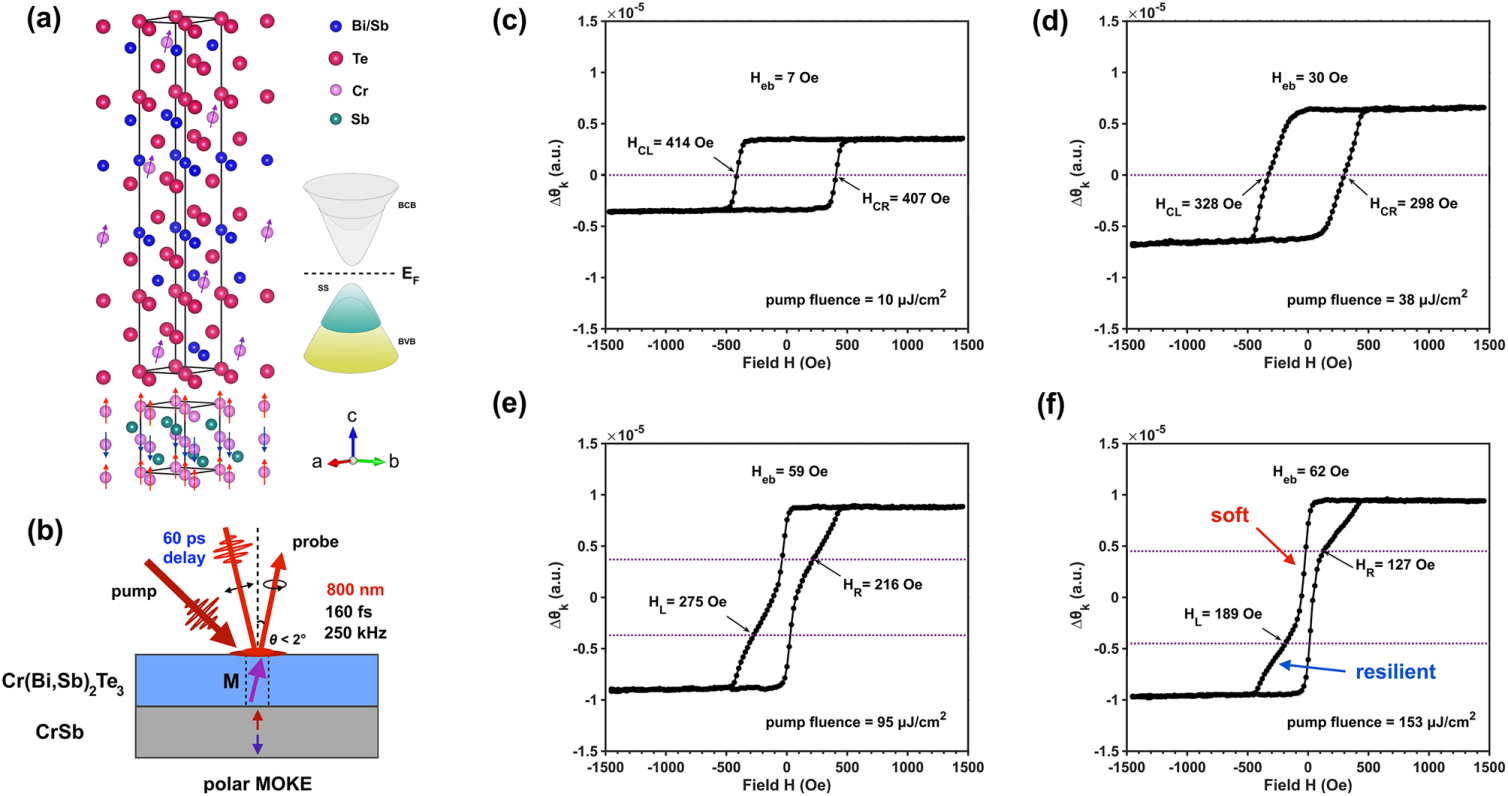
Crystal structure, experimental schematic, and pump-modulated MOKE loops in MTI/AFM heterostructure. (**a**) The crystal structure and atomic moments near the MTI/AFM interface. Cr atoms with their magnetic moment shown in red and blue are those belonging to the CrSb layer at the interface and exhibit out-of-plane AF order. Magnetic moments denoted as purple arrows are associated with doped Cr atoms in the MTI layer, which show long-range out-of-plane FM order. (**b**) Schematic of the pump-modulated MOKE measurements. The transient polar MOKE configuration probes the magnetization change in the perpendicular direction. (**c**–**f**) pump-modulated MOKE hysteresis loops with a variety of pump fluences. The loop transforms from a uniform shape at the lowest pump fluence (**c**) to an exchange-spring shape at the highest pump fluence (**f**), which manifests the development of photoinduced bulk-related and surface-related ferromagnetism. The exchange bias effect becomes prominent as the pump fluence increases.

The crystal structure of Cr-(Bi,Sb)_2_Te_3_/CrSb heterostructure is depicted in Fig. 1a, where the magnetic moments of Cr atoms are denoted by arrows. While the Cr-(Bi,Sb)_2_Te_3_ layer can sustain long-range magnetic order at temperatures below ~ 50 K, the ferromagnetism of the MTI layer is greatly enhanced by interfacial exchange coupling to the AFM CrSb layer; enabling the MTI magnetism to persist at temperatures in excess of 100 K that may be more easily accessed by optical experiments^5^. The static magnetic properties of the Cr-(Bi,Sb)_2_Te_3_/CrSb heterostructure are studied by polar MOKE measurements (see Method and Supplementary). Hysteresis loops taken at 78 K display perfect squareness following the subtraction of a linear background, indicating a well-developed ferromagnetic order with strong PMA. The corresponding enhanced MTI Curie temperature revealed by our static MOKE measurements is around 120 K, significantly enhanced when compared with a bare MTI layer with similar magnetic doping levels^5^. Due to the PMA of these samples, the effective magnetization of the MTI layer is assumed to be only slightly canted with respect to the surface normal (z-direction) under the applied external field.

Pump-modulated MOKE measurements were subsequently performed to investigate the photo-induced magnetic dynamics in the heterostructure. A schematic diagram of the experimental geometry is illustrated in Fig. 1b. The probe light is incident at a small angle of less than 2° to the film normal, sensing the magnetic response in the polar direction. A pump beam of a much larger radius is focused onto the sample; illuminating the area sensed by the probe beam. By chopping the pump beam and measuring the AC response at the photodetector using a lock-in technique (see Method), these experiments directly capture photo-induced changes in magnetization rather than the absolute Kerr rotation. As a consequence, unlike their static counterparts, the transient MOKE loops do not exhibit a slant background. Figure 1c–f shows the transient MOKE loops modulated by p-polarized pump light of different excitation fluences $I$ with a fixed pump-probe delay of 60 ps. With the lowest pump fluence $I=$ 10 µJ/cm^2^ (Fig. 1c), the transient MOKE loop maintains a perfect squareness indicating a uniform magnetization in the MTI layer. As we increase the pump fluence to 38 µJ/cm^2^ (Fig. 1d), the transient MOKE signal is enhanced, while broadening of the magnetic switching at the coercive field suggests a softening magnetic order with a significantly reduced coercivity. Finally, when the excitation fluence is increased to 95 µJ/cm^2^ (Fig. 1e) and beyond (Fig. 1f), we observe a qualitative transition in the shape of the MOKE hysteresis loop. While measurements conducted with relatively low pump fluence produce transient signals with sharp switching features, measurements taken at high pump fluences instead display two-step magnetization switching, suggesting different regions of the sample experience distinct coercive fields^27,28^. In the two-step hysteresis loops it is salient that one part narrows sharply, displaying a dramatically reduced coercivity of 33 Oe, while the remaining switching signal is comparatively resilient with a saturation field of approximately 430 Oe. Such a dynamical magnetic structure is analogous to a dynamical exchange-spring “hard-soft” bilayer set.

**Figure 2:**
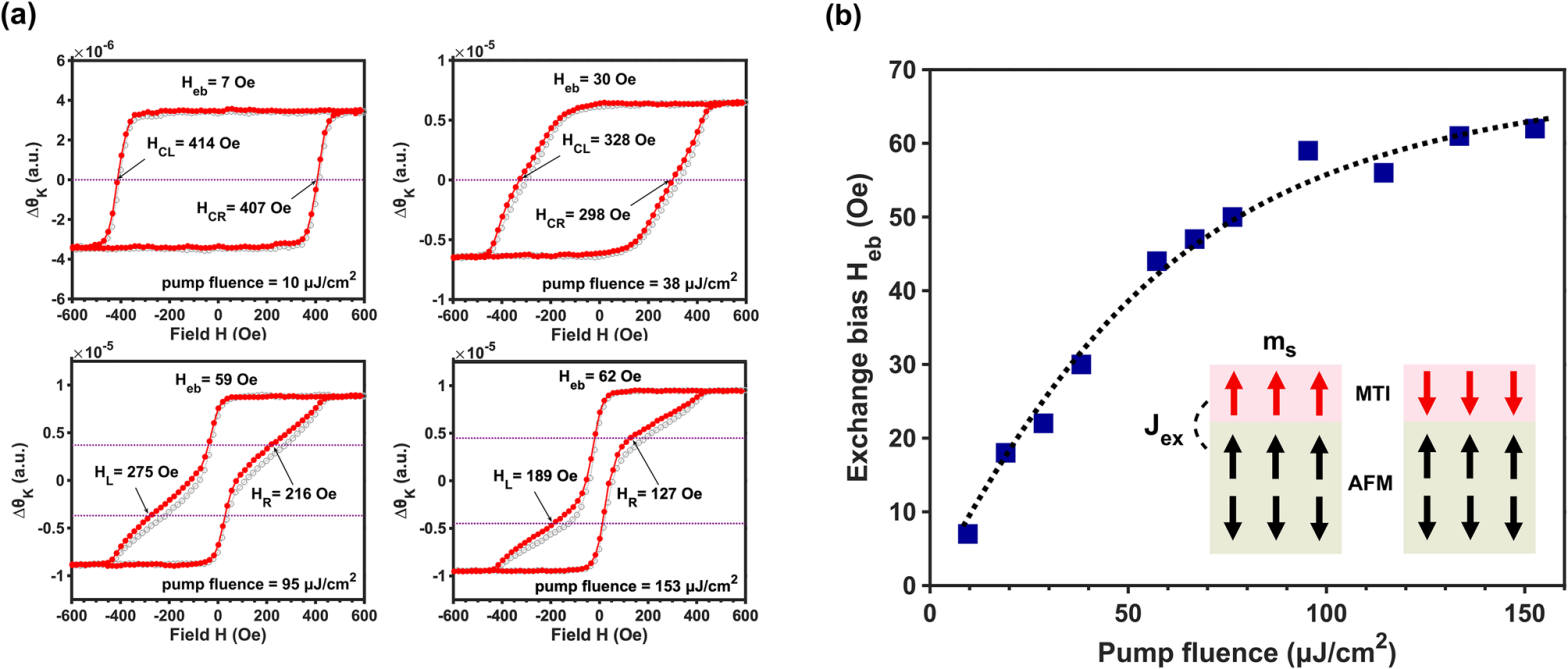
Pump-fluence dependent magnetization dynamics and exchange bias effect. (**a**) exchange bias fields extracted from various pump-modulated MOKE hysteresis loops, where red dots and gray circles are original and center-flipped data, respectively. (**b**) exchange bias fields *H*_eb_ as a function of pump fluence. The solid circles are the experimental data, and the dashed line is artificial for visual guidance. The insert schematic diagram illustrates the photoinduced exchange bias effect where the red arrows represent the photoinduced spin polarization at the surface and the black arrows correspond to the spin texture in the CrSb layer.

In addition to the evolving exchange-spring behavior presented above, an unusual, photo-induced exchange-bias effect is also observed at high pump fluence. This effect is apparent in Fig. 2a, which presents two transient MOKE loops: one representing an unprocessed signal, and the other generated by rotating the raw data by 180° around the origin. By presenting the data in this fashion, asymmetry between the positive and negative magnetic switching distribution function is easier to visualize. Those transient loops collected using higher pump fluence very clearly do not overlap is indicative of an incipient exchange bias effect with increasing pump power. Furthermore, this exchange bias is not uniformly distributed in all the loops, rather, it is most pronounced in the region of the hysteresis loop displaying the larger coercive field. To elucidate the pump dependency of this effect, we show the magnitude of the maximum exchange-bias field as a function of pump fluence in Fig. 2b. The dynamical exchange-bias field *H*_eb_ reaches a plateau of around 60 Oe after the initial increase from 7 Oe.

## Discussion

The pump-modulated MOKE results (Fig. 1c–f) show the evolution of magnetism in a Cr-BST/CrSb bilayer sample from a hard, homogenous ferromagnetic state, into apparently one composed of two distinct magnetic components; one component exhibiting a relatively small coercive field and the other featuring both a prominent exchange bias and a large coercive field. We propose that when subjected to high pump intensities, the normally well-coupled surface and bulk magnetic order of the MTI decouple, where the magnetic switching that occurs at large coercive fields originates from the MTI/CrSb interface and switching observed at small-coercive fields corresponds to signals derived from the bulk of the MTI layer as explained in the following.

When radiated upon the material, the pump beam perturbs the magnetic order through several distinct channels. The shortest-lived effect is a temporary excitation of the electronic system into a highly non-thermal distribution. This effect occurs almost instantaneously and leads to an ultrafast demagnetization of the sample. Through a combination of electron–electron and electron–phonon processes, the electrons rapidly relax into an approximately thermal Fermi–Dirac distribution over the first few hundred fs to 1 ps^29–32^. As the electronic system returns to a thermal Fermi–Dirac distribution, it features an elevated density of photo-excited quasiparticles, that, due to a bottleneck in the density of states at the Dirac point in the MTI, may take hundreds of ps or longer to fully return to their equilibrium state^31,32^. These photo-induced carriers in turn influence the magnetic properties of the MTI system. Finally, and most trivially, the pump beam will transfer energy to the crystal lattice. The energy imparted into the lattice will raise the temperature of the sample beyond that of the cryogenic bath. This final effect is sufficiently long-lived it is unlikely to recover on the time scales accessible by the time-delay stage (~ 1 ns). It is noteworthy that the electron temperature may not match that anticipated by the lattice temperature after the electronic system reaches to a thermal Fermi–Dirac distribution.

**Figure 3:**
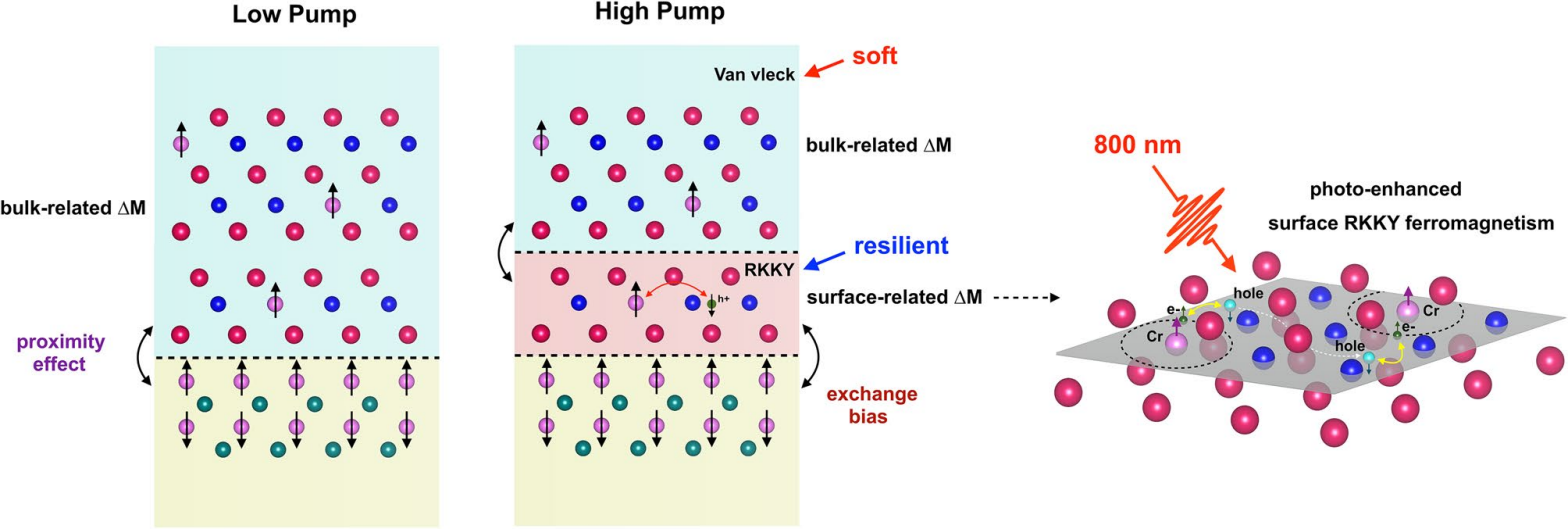
Divergent photo-induced effects on surface-related and bulk-related ferromagnetism. At low pump fluences, the photo-induced transient magnetism shows a uniform bulk-related magnetization. At high pump fluences, the photo-induced magnetization exhibits a weakened bulk-related magnetization and a resilient surface-related magnetization. The surface spin coupling is enhanced by the photoexcited Dirac holes with opposite spin polarization.

**Figure 4:**
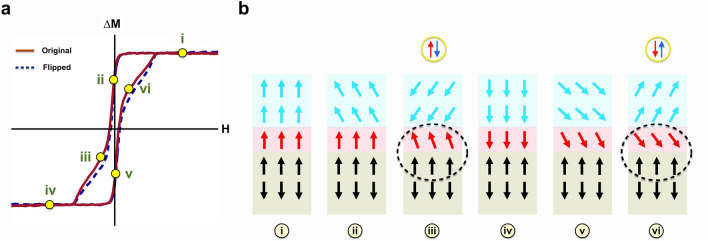
Schematic of the coupling model of the surface- and bulk-related ferromagnetic phases in Cr-doped (Bi,Sb)_2_Te_3_/CrSb heterostructure. (**a**) the red curve is the original data, and the blue curve is the same data flipped regarding x and y axis, which highlights the photo-induced exchange bias effect. (**b**) the switching process of the photo-induced magnetization in the Cr-doped (Bi,Sb)_2_Te_3_ layer. The red and blue arrows correspond to the photo-induced spin polarization corresponding to transient surface- and bulk-related ferromagnetism, respectively. The canted spin “arrows” are shown for simplicity of a possible multi-domain switching fashion. As illustrated in (iii) and (vi), when the direction of the photo-induced bulk-related spin polarization is reversed under a small external field, the photo-induced surface-related magnetization resists the switching due to stronger magnetism. Consequently, a photo-induced antiparallel spin configuration of the two ferromagnetic phases is formed. On the other hand, the interfacial exchange coupling between the MTI surface layer and the CrSb layer results in a photo-induced exchange bias field as the CrSb spins keep the photo-induced MTI surface spins in their original orientations in an FM alignment.

In the Cr-(Bi,Sb)_2_Te_3_/CrSb bilayer system, the surface-state magnetization in the MTI layer is predominately mediated by the RKKY interaction between Dirac holes and impurity spins, while the bulk-state magnetization originates from the long-range Van Vleck *p-d* spin coupling among local moments. When the pump fluence is low, the surface RKKY exchange interaction is relatively weak, and the laser-induced thermal effect is small. Correspondingly, the magnetic moments at the surface and in the bulk are well coupled with each other. When the pump fluence is increased, the photo-induced carrier density and lattice heating both become more pronounced. While the elevated lattice temperature is inhospitable to magnetism of both RKKY and van Vleck origins, the impact of the photo-injected holes at the surface Dirac bands is more complex. van Vleck mediated bulk magnetism, being free-carrier agnostic, is insensitive to the increased density of excited carriers. At the surface, the elevated carrier density can strengthen the RKKY interaction and partially compensate for the deleterious effects of the elevated temperature. Macroscopically, the divergent photo-response of the RKKY (surface) and van Vleck (bulk) mediated magnetic moments (Fig. 3) leads to a mismatch in their respective coercive fields, with the bulk magnetic moments displaying a reduced coercivity compared with the surface. Figure 4 schematically shows a model of the photo-induced transient magnetic state in the MTI Cr_0.26_(Bi_0.3_Sb_0.7_)_1.74_Te_3_ under external field switching. The narrow component of the transient coercive field measured at the highest pump fluence is similar to what is observed in the 120-K MOKE loop collected with no pump (see Supplementary); suggesting that the film temperature may experience a temporary increase of nearly 40 K. This observation is consistent with the temperature rise we predict using the known heat capacity and optical absorption characteristics of these films (see Supplementary), although the number is not intended to be presented as an accurate temperature.

To corroborate the understanding that the regions of large coercive fields correspond to the MTI surface magnetism, we now consider the photo-induced exchange-bias effect which is related to the exchange energy of the MTI/AFM interface. The onset of perpendicular exchange bias is a common feature of proximity coupled antiferromagnet/ferromagnet bilayers. As magnetic exchange bias is a purely interfacial phenomenon, the fact that the exchange bias is most pronounced in the region of the hysteresis loop featuring the large coercive field immediately suggests this signal indeed originates at the surface of the topological insulator. Meanwhile, the absence of exchange bias within the low-field switching feature indicates a decoupling of this interfacial magnetism from the bulk of the MTI. The increasing exchange bias observed at large pump intensities can be further understood using Meiklejohn and Bean’s exchange anisotropy model^33,34^. Based upon this model, the macroscopic bias field *H*_eb_ can be expressed with the Heisenberg-like interface exchange energy as ${H}_{eb}=\frac{{J}_{eb}{S}_{CrSb}{S}_{MTI}}{{a}^{2}{M}_{MTI}{t}_{MTI}}\mathrm{cos}\theta$ where *J*_eb_ is the effective interfacial exchange energy, ${S}_{CrSb}$(${S}_{MTI}$) is the spin of an individual layer of CrSb (MTI) at the interface, $\theta$ is the angle between the applied field and perpendicular direction of the thin films, ${a}^{2}$ is the unit cell area and ${t}_{MTI}$ is the effective thickness of MTI surface layers. The pump beam is unlikely to significantly modify intrinsic features of the bilayer system such as the Cr spin state, the exchange constant, or unit cell area. However, the pump-driven decoupling of the surface and bulk reduces the effective thickness and magnetization of the MTI surface layer, increasing the effective exchange-bias field.

**Figure 5:**
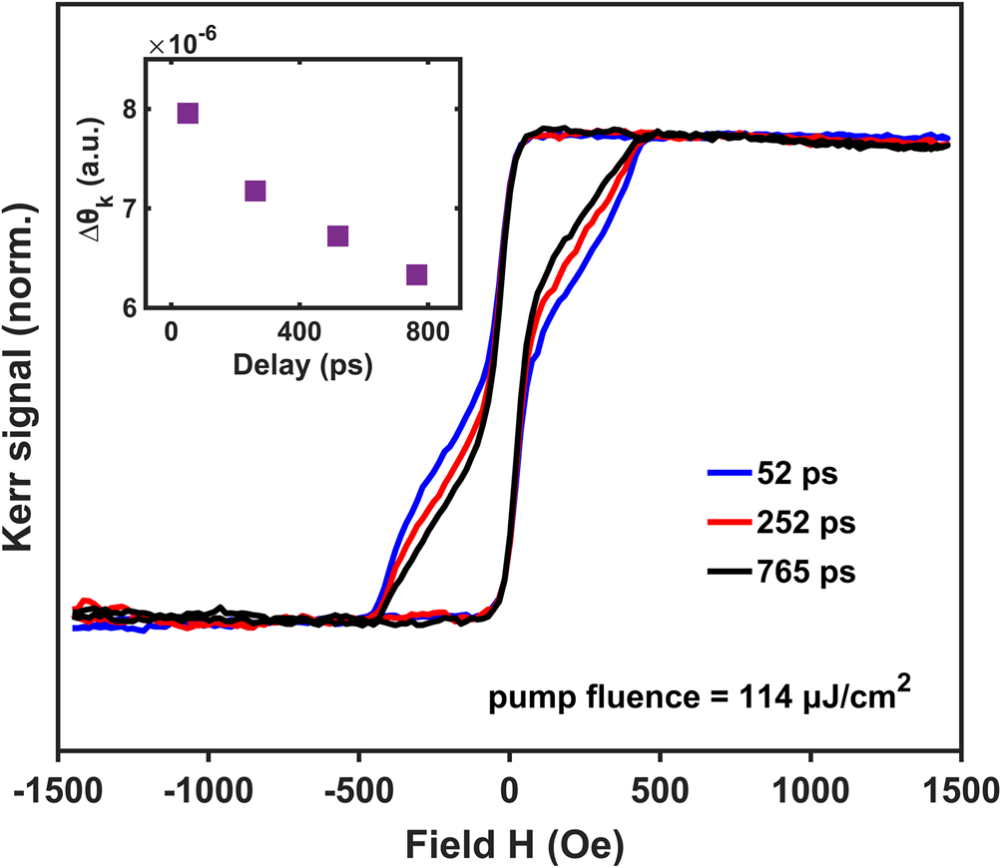
Pump-modulated MOKE loops in time domain. Pump-modulated MOKE loops at $I=$ 114 µJ/cm^2^ and $H=$ 1452 Oe at different pump-probe delays. The insert shows the magnitude of magnetization enhancement as a function of pump-probe delay (error bars are < 2%).

Transient MOKE loops collected at different pump-probe delays provide further support for a pump-driven decoupling of the MTI surface and bulk magnetism, wherein the latter is uniformly suppressed through thermal effects and the former is sustained by optically excited carriers. As observed in Fig. 5, with increasing time delays the switching feature observed at large coercive fields becomes far less pronounced. Notably, rather than recover the square-shaped large coercivity switching observed at low pump intensities, with increasing time delays the coercivity of the transient features continues to decrease, moving further from, rather than closer to the expected equilibrium behavior. This counter-intuitive development indicates that the mechanism sustaining the large coercive field region of the two-step switching has a short lifetime on the order of hundreds of ps, while its small coercive field counterpart is comparatively long-lived. As discussed above, the timescales associated with these two switching behaviors notably correspond with the known characteristic times of carrier relaxation and thermal dissipation in these materials; indicating the large coercive field region is electronic in origin, whereas the small coercive field region is maintained by long-term thermal effects.

Based upon the above qualitative understanding, we estimate the exchange energy *J*_eb_ at the MTI/AFM interface. In our highest-pump fluence case, with ${t}_{MTI}$ = 0.43 nm being the thickness of three monolayers in the vicinity of AFM and ${\text{M}}_{\text{MTI}}$ = 50 emu/cm^3^ being the MTI’s saturated magnetization^5^, we obtain the interfacial energy $\sigma =1 \times {10}^{-4}$ erg/cm^2^ from $\sigma =$ ${{\text{H}}_{\text{eb}}{\text{M}}}_{\text{MTI}}{{\text{t}}}_{\text{MTI}}$. With ${S}_{AFM}$ = $\frac{3}{{2}}$ and $S$ = $\frac{3}{{2}}$, the interlayer exchange coupling parameter is estimated as *J*_eb_ = 1 × 10^−19^ erg. While the magnitude of the exchange bias field observed here is comparable to what has been previously reported in the Cr_2_O_3_/CBST system^35^, the size of *J*_eb_ is three orders of magnitude smaller than typical perpendicularly exchange-biased FM/AFM systems such as [Pt/Co]/Cr_2_O_3_ and [Pt/Co]/IrMn, where the exchange couplings are believed to be short-range interactions^36,37^.

In summary, we have studied the magnetization dynamics of Cr_0.26_(Bi_0.3_Sb_0.7_)_1.74_Te_3_/CrSb bilayer system using ultrafast magneto-optical techniques. We manipulate the magnetizations by pump laser irradiation and observe a photo-induced dynamical exchange-spring magnetic structure from the pump-modulated MOKE results. We identify the two distinct photo-induced transient ferromagnetic phases as originating from the surface and bulk of the MTI. The soft ferromagnetic phase corresponds with the photo-induced bulk-magnetism while the resilient ferromagnetic phase relates to the photo-induced surface-magnetism. We then estimate the interfacial exchange energy and compare it with other exchange-biased FM/AFM systems. The results are relevant to other MTI-based bulk, surface, and interface systems with carrier-dependent and carrier-independent exchange coupling effects. Our findings facilitate the practical applications of MTI and MTI/AFM systems in laser-assisted magnetic recording, ultrafast optoelectronics, and spintronics.

## Method

### Sample fabrication

The Cr_y_(Bi_x_Sb_1-x_)_2-y_Te_3_/CrSb heterostructure investigated in this work was fabricated in an ultrahigh vacuum molecular beam epitaxy (MBE) system. The samples were fabricated on epi-ready semi-insulating GaAs(111)B substrates with oxide-desorption processes carried out at 580 °C for 30 min in advance. The substrate temperature was kept at 200 °C for the rest of the growing process. Bi and Cr were evaporated from standard Knudsen cells, while Sb, Se, and Te were evaporated by standard thermal cracker cells. The concentration *x* of Bi and dopant concentration y of Cr are controlled to 0.3 and 0.26, respectively. Real-time reflection high-energy electron diffraction (RHEED) was used to monitor all the growth cycles. RHEED patterns were optimized to very sharp, smooth, streaky patterns, while the intensity oscillations were used to calibrate growth rates. To avoid the three-dimensional growth mode of CrSb with low crystal quality, a 2-nm buffer layer of undoped (Bi, Sb)_2_Te_3_ is grown firstly on the GaAs substrate before the growth of CrSb. After the film growth, a 2 nm Al layer was evaporated on the sample surface in situ at room temperature to prevent contamination and oxidation.

### Static MOKE measurements

In the static MOKE studies, we measured the FM magnetization of the MTI layer by irradiating the sample with s-polarized light and probing the p-component of the reflected light with a photodiode. The 800-nm probe light is generated by a Ti/sapphire amplifier laser system with 150-fs pulses at 800-nm wavelength and a repetition rate of 250 kHz. The probe beam is modulated by a chopper along with the integration of a lock-in amplifier. The incident light has a small angle of less than 2° to the film normal, which probes the magnetic response in the perpendicular direction. The external magnetic field is applied with an estimated angle of 20° to the sample’s surface with a magnetic field range of ± 1815 Oe. The sample was sealed under a high vacuum environment in the cryostat during the experiment.

### Pump-modulated MOKE measurements

We performed pump-modulated MOKE measurements in a pump-probe setup with various intensities of pump lights. The probe utilizes the MOKE technique with crossed polarizers to investigate the transient magnetization change along the out-of-plane direction, with the same configuration of the static MOKE measurements. The pump pulses are incident to the sample surface with an angle of 25° regarding film normal, and a fixed pump-probe delay of 60 ps is chosen across the pump-fluence-dependent measurements. The direction of the applied field keeps the same as the static MOKE case. For the time-dependent measurements, additional pump-probe delays of 270 ps and 770 ps are set to study the evolution of pump-modulated MOKE in the time domain. The pump beam is focused to a spot ~ 0.4 mm in diameter and modulated by a chopper with a frequency of around 1800 Hz to alternate the intensity of the probe beam at the modulated frequency. The focus area of the probe beam is ~ 0.2 mm in diameter and centered in the focus spot of the pump beam. The applied external field in these measurements is ± 1452 Oe.

## Supplementary Information

Supplementary Information.

## Data Availability

The datasets generated during and/or analyzed during the current study are available from the corresponding author upon reasonable request.
