# Supplementary material for: Ultrafast optical control of surface and bulk magnetism in magnetic topological insulator/antiferromagnet heterostructure
Source: Sci Rep. 2022 Jul 15;12:12117. doi: 10.1038/s41598-022-16205-3 (PMC9287552; doi:10.1038/s41598-022-16205-3)
Supplement: Supplementary file 1 — Supplementary Information. [file 41598_2022_16205_MOESM1_ESM.docx]

**Supplementary:** Ultrafast Optical Control of Surface and Bulk Magnetism in Magnetic Topological Insulator/Antiferromagnet Heterostructure

Peiwen Liu^1^, Chris Eckberg^2,3,4,5^, Lei Pan^2^, Peng Zhang^2^, Kang L. Wang^2^ and Gunter Lüpke^1,^*

^1^ Department of Applied Science, The College of William and Mary, Williamsburg, Virginia 23187, USA

^2^ Department of Electrical Engineering, University of California, Los Angeles, California 90095, USA.

^3^ Fibertek Inc., Herndon, VA 20171

^4^ DEVCOM Army Research Laboratory, Adelphi, Maryland 20783

^5^ DEVCOM Army Research Laboratory, Playa Vista, California 90094

*email: [gxluep@wm.edu](mailto:gxluep@wm.edu)

**Temperature-dependent static MOKE results**

The static magnetic properties of the Cr-(Bi,Sb)_2_Te_3_/CrSb heterostructures are studied by polar magneto-optical Kerr effect (MOKE) measurements with applied magnetic field canted 20$^{\circ}$ from the film surface normal (Fig. S1). The static MOKE hysteresis loops show perfect squareness at 78 K after subtraction of the slanted background signal, indicating a well-developed ferromagnetic order of the MTI layer. The slanted background signal may originate from the CrSb layer. Under the relatively low external field (< 0.2 T) and due to the large proximity effect with the CrSb layer, the effective magnetization of the MTI layer, which has rugged perpendicular anisotropy, is only slightly canted from the out-of-plane direction. As shown in Fig. S1, the ferromagnetic ordering of MTI layer disappears at a temperature of 120 K to 140 K.

**
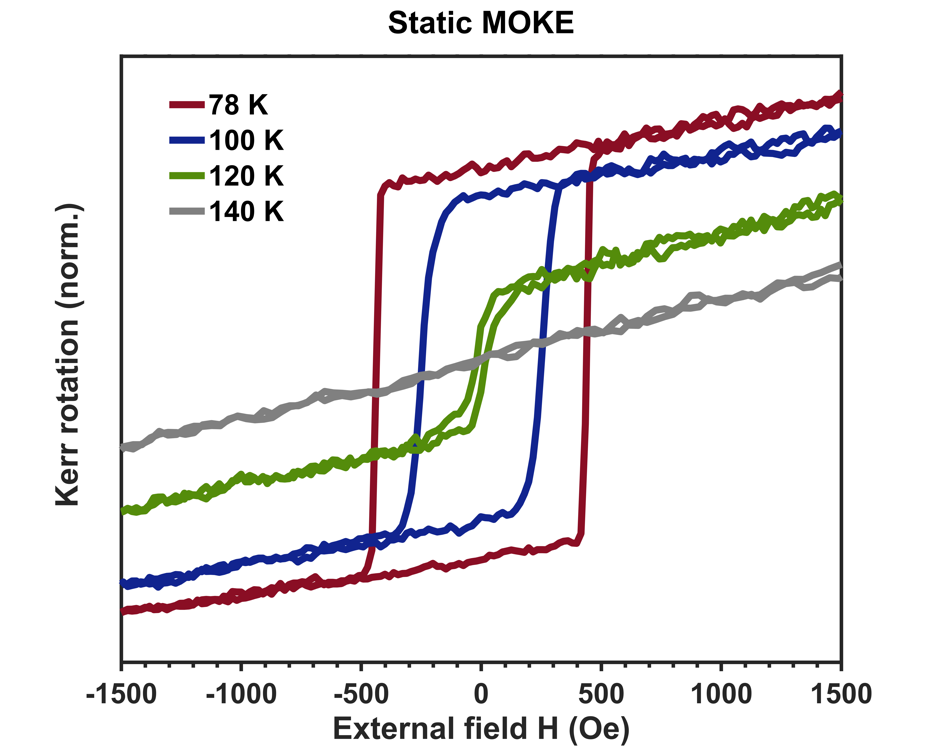
**

**Figure S1 | Static MOKE data.** Temperature dependent hysteresis loops obtained with static MOKE measurements. The results are normalized with respect to the 78 K data.

**Pump-modulated MOKE results**

The pump-modulated MOKE results are shown in Fig. S2 with pump fluences from 10 $-$153 $\mu$J/cm^2^. The static MOKE signal for comparison is $\text{1.5}\text{×}\text{10}^{\text{-}\text{5}}$ mV. When the pump fluence reaches 67 $\mu$J/cm^2^, the MOKE signal starts to saturate and an exchange-spring magnetization behavior is revealed. The dynamical magnetization enhancement is valued by the magnitude of transient MOKE loops, which is then combined with the static MOKE signal for the quantitive analysis. The data of dynamical exchange bias effect are obtained by extracting the maximum field offsets of the original the flipped loops. The magnitude of the pump-induced Kerr signal as a function of pump fluence is plotted in Fig. S3. While the transient Kerr signal increases monotonically with increasing pump fluence, at higher pump intensities, the increasing tendency retards and the change of Kerr signal approaches a level of saturation.


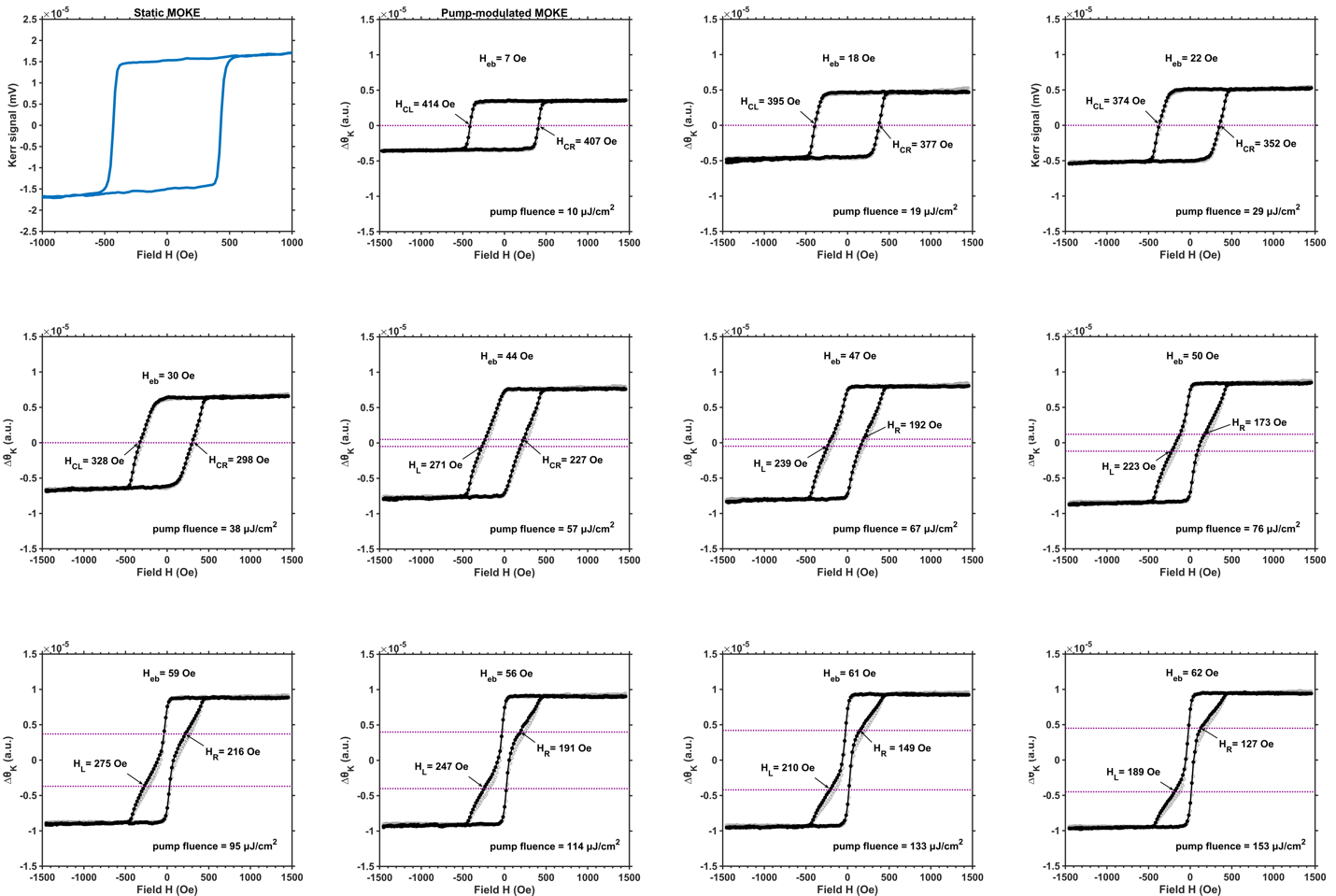


**Figure S2 | Pump-fluence dependent transient MOKE data.** Pump-modulated MOKE hysteresis loops with a variety of pump fluences and the static MOKE loop with the same experimental configuration. The loop develops from a uniform shape at the lower pump fluences to an exchange-spring shape at the higher pump fluences**.** The dashed purple lines indicate where the maximum exchange biased field is extracted.


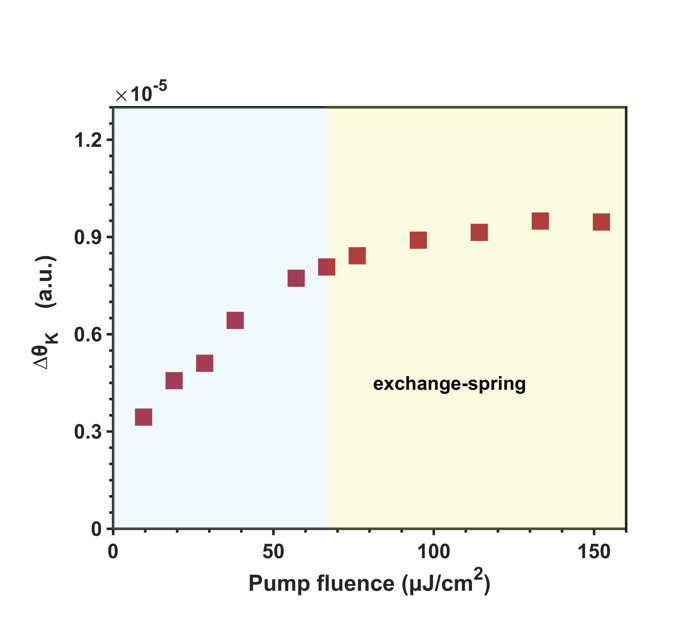


**Figure S3 | Pump-fluence dependent transient MOKE strength.** Magnitude of transient MOKE signal as a function of pump fluence. The solid squares are the heights of pump-modulated MOKE loops. The yellow shade corresponds to the region of exchange-spring hysteresis loops.

**Pump fluence dependent time-resolved MOKE**

Figure S4(a) shows the time traces of the normalized pump-modulated MOKE signals at various pump fluences and we observe that the initial rise times don’t change considerably for all pump fluences. Figure S4(b) shows the corresponding recovery times based on a two-lifetime model, $I=I_{s}e^{-t/\tau_{s}}+I_{l}e^{-t/\tau_{l}}$, where $I_{s}$ ($I_{l}$) and $\tau_{s}$ ($\tau_{l}$) are the faster (slower) recovering signal and characteristic times, respectively. The slower recovering time $\tau_{l}$ barely changes from 19.1 $\text{μ}$*J*/cm^2^ to 95.4 $\text{μ}$*J*/cm^2^ and increases by a factor of two at 127 $\text{μ}$*J*/cm^2^. Figure S4(a) displays the time-resolved data of reflectivity change $\Delta R/R$ (s-in s-out polarization configuration) at a pump fluence of 153$\text{ }\text{μ}$*J*/cm^2^, where the rise time is similar to those of the time-resolved MOKE signals, but the recovery time (~ 1 ns) is much shorter. It should be noted that in Figure S4(a), both magnetization change and reflectivity change contribute to the observed signals, although the incoming and outgoing beam is cross-polarized by polarizers with a small angle deviation to increase the signal-to-noise ratio of MOKE data. Nevertheless, after the rising peak, the intensity of the nonmagnetic signal in Figure S4(a) is one order of magnitude smaller than that of the total signal, manifesting that in the decaying stage, the magnetization dynamics dominate the time-resolved signals.


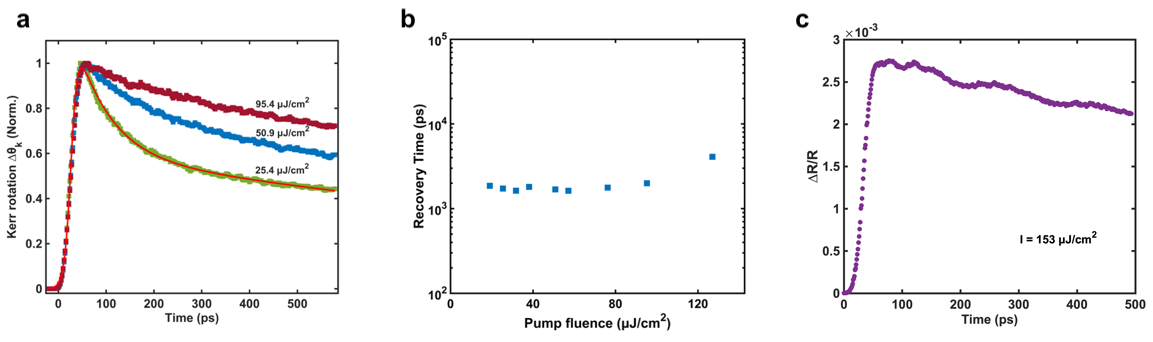


**Figure S4 | Pump-fluence dependent time-resolved MOKE results. a,** transient MOKE signals as a function of pump-probe time delay. The solid squares are measured data and the red line is the simulation of the data. **b,** the extracted slower recovery characteristic times as a function of pump fluences. **c,** ultrafast time trace of reflectivity change. The solid circles are the experimental data collected at the same temperature with time-resolved MOKE measurements, 78 K.

**Estimation of the thermal effect and photoinjected carrier density**

The 2nm thin Al_2_O_3_ cap-layer with a high transmission at an 800-nm wavelength (greater than 95%) is considered to be transparent. The estimated reflection of Sb_2_Te_3_ single crystal is $R$ = 50%.^[1,2]^ The estimated extinction coefficient for Sb_2_Te_3_ thin film is $\kappa$ = 3, ^[3,4]^ and the absorption coefficient at 800 nm is derived as $\alpha=\frac{4\pi\kappa}{\lambda}$ ~ 0.047 nm^-1^. The laser system delivers pulses with an energy of 4 $\mu J$ at 1000-mW power output and a repetition rate of 250 kHz therefore the pump energy is 0.192 $\mu J$ with the maximum power output of 48 mW irradiating on the sample. The effective pump beam radius is $r$ = 0.2 mm and the maximum pump fluence is 153 $\mu$J/cm^2^. Within the first several hundred femtoseconds or first few picoseconds after the laser pulse, electrons are excited to a high temperature and then the energy is transferred to the lattice through electron-electron and electron-phonon scattering. The estimation of the temperature increase is given by

$$(1-R)\cdot I\cdot A\cdot\alpha\int_{0}^{D} e^{-\alpha x}dx{=\bar{\text{C}}}_{p}\frac{\rho Ad}{\text{M}}\Delta T$$

where *D* is the length of absorption, $\rho$ = 6.5 g/cm^3^ and $\text{M}$ = 626 is the density and molecular weight of Sb_2_Te_3,_ respectively, ${\bar{\text{C}}}_{p}=$ 110 J∙mol^-1^K^-^1 is the averaged special heat capacity, and $A=\pi r^{2}$ is the effective irradiation area. the limits of a zero and full reflection of light passing through the MTI layer, the estimated increase Considering of temperature with a pump fluence of 153 $\mu$J/cm^2^ is in the range of $\Delta\text{T}$ = 26 $-$ 42 K with *D* = 10 nm and 20 nm correspondingly. Next, the density of holes generated by photons absorbed in the MTI surface layer is estimated by

$$(1-R)\cdot\alpha\int_{d-\Delta l}^{L} e^{-\alpha x}dx\cdot I=\Delta\text{n}\text{ℏω}$$

where $d$ = 10 nm is the thickness of the MTI layer, $\Delta l$= 0.43 nm is the thickness of surface-state layers and $\text{ℏω}$ = 1.55 eV is the photon energy. Similarly, with *L = d* and *L = d+*$\Delta l$, the estimated photoexcited surface hole density is $\text{n}_{c}\text{ }\text{= }\text{4}\text{×}\text{10}^{\text{12}}-\text{ }\text{8}\text{×}\text{10}^{\text{12}}\text{/}\text{cm}^{\text{2}}$ with a pump fluence of 153 $\mu$J/cm^2^.

**Reproducibility of pump-modulated MOKE measurements**

The same pump-modulated MOKE measurements taken after the highest pump fluence experiment show similar results which indicates that the sample has not been damaged by the pump excitations in our study.

**References**

1. Shaik, M. & Motaleb, I. A. Investigation of the optical properties of PLD-grown Bi_2_Te_3_ and Sb_2_Te_3_. *IEEE EIT 2013* (2013).
2. Sobolev, V. V. *et al*. Reflectivity Spectra of the Rhombohedral Crystals Bi_2_Te_3_, Bi_2_Se_3_, and Sb_2_Te_3_ over the Range from 0.7 to 12.5 eV. *Phys. Status Solidi B* **30**, 349–355 (1968).
3. Park, J.-W. *et al*. Optical properties of (GeTe, Sb2Te3) pseudobinary thin films studied with spectroscopic ellipsometry. *Appl. Phys. Lett.* **93**, 021914 (2008).
4. Lawal, A. *et al*. Sb_2_Te_3_ crystal a potential absorber material for broadband photodetector: A first-principles study. *Results Phys*. **7**, 2302–2310 (2017).
